# National Convention for the Revision of the United States Pharmacopœia

**Published:** 1869-11

**Authors:** 


### National Convention for the Revision of the United States Pharmacopœia.

The President of the last Pharmaceutical Convention for the revision of the U. S. Pharmacopœia, announces the following resolutions adopted at that time, respecting the coming decennial revision of the same:

"1. The President of this Convention shall on the first day of May, 1869, issue a notice, requesting the several incorporated Medical Societies, the incorporated Medical Colleges, the incorporated Colleges of Physicians and Surgeons, and the incorporated Colleges of Pharmacy throughout the United States, to elect a number of delegates not exceeding three, to attend a general convention to be held at Washington, on the first Wednesday in May, 1870.

"2. The several incorporated bodies, thus addressed, shall also be requested by the President to submit the pharmacopoeia to a careful revision, and to transmit the results of their labors, through their delegates, or through any other channel to the next convention.

"3. The several Medical and Pharmaceutical bodies, shall be further requested to transmit to the President of this Convention, the names and residents of their respective delegates, as soon as they shall have been appointed, a list of whom shall be published, under his authority, for the information of the Medical Public, in the newspapers and medical journals in the month of March, 1870."

In compliance with the above resolution, the President of the convention announces that a meeting will be held in Washington, D. C., on the first Wednesday in May, 1870, and requests that the several incorporated bodies shall after a revision of the United States Pharmacopoeia, send the results of their labors to the Convention, and further requests that they transmit to the President the names and residences of their several delegates as soon as elected, that the list may be published.

GEO. B. WOOD,
President of the Convention of 1860.

